# State-change decisions and dorsomedial prefrontal cortex: the importance of time

**DOI:** 10.1016/j.cobeha.2018.06.017

**Published:** 2018-08

**Authors:** Nils Kolling, Jill X O'Reilly

**Affiliations:** 1 Wellcome Integrative Neuroimaging (WIN), Department of Experimental Psychology, University of Oxford, Oxford, UK; 2 Oxford Centre of Human Brain Activity, Wellcome Centre for Integrative Neuroimaging, Department of Psychiatry, University of Oxford, Oxford, UK; 3 Wellcome Integrative Neuroimaging (WIN), Centre for Functional MRI of the Brain (MRI), Nuffield Department of Clinical Neurosciences, John Radcliffe Hospital, University of Oxford, UK; 4 Donders Institute for Brain, Cognition and Behaviour, Radboud University, Nijmegen, Netherlands

## Abstract

Different kinds of decision making can be categorized by their differential effect on the agent’s current and future states as well as the computational challenges they pose. Here, we draw a distinction between within-state and state-change decision-making, and propose that a dedicated decision mechanism exists in dorsomedial prefrontal cortex (dmPFC) that is specialized for state-change decisions. We set out a formal framework in which state change decisions may be made on the basis of the integrated momentary reward rate, over the intended time to be spent in a state. A key feature of this framework is that reward rate is expressed as a function of continuous time. We argue that dmPFC is suited for this type of decision making partly due to its ability to track the passage of time. This proposed function of dmPFC is placed in contrast to other evaluative systems such as the orbitofrontal cortex, which is important for careful deliberation within a specific model-space or option-space and within a decision strategy.

**Current Opinion in Behavioral Sciences** 2018, **22**:152–160

This review comes from a themed issue on **Apathy and motivation**

Edited by **Christopher Pryce** and **Masud Husain**

For a complete overview see the Issue and the Editorial

Available online 25th July 2018

**https://doi.org/10.1016/j.cobeha.2018.06.017**

2352-1546/© 2018 The Authors. Published by Elsevier Ltd. This is an open access article under the CC BY license (http://creativecommons.org/licenses/by/4.0/).

Within the field of decision-making, two complementary classes of problems may be defined. Firstly, at any given moment, agents must select the best action from those available, given the circumstances or state in which they find themselves. But a second, higher order problem is the need to evaluate the current state, or set of available actions, as a whole and thus decide whether to persist in that state or try to change it. We will argue in this review that these types of decisions, which we call *within-state* and *state-change* decisions respectively, pose different computational problems and are mediated by different regions of frontal cortex. Focussing on state-change decisions, we will argue that the representation of time is a key factor in the evaluation of current and possible states, and that the medial prefrontal cortex, including pre-SMA and anterior cingulate cortex, is well placed to make such decisions.

## Within-state decisions vs state-change decisions

For the purposes of this review, we define the term *global state* in terms of the cognitive map. The cognitive map is a structured neural representation of the probabilistic relationships between stimuli, actions and outcomes [1]. A useful distinction can then be drawn between *within-state* decisions, in which the agent makes decisions based on the relative values of actions within the current cognitive map, and *state-change* decisions, in which the agent evaluates the *total* value of remaining in the current environment or global state vs the value of removing itself to a different environment with different stimuli, actions and outcomes.

In laboratory paradigms, *within-state* and *state-change* decisions can be separated in a relatively clear-cut way by ensuring that participants do not know what the outcome of changing their state will be — thus the potentially changed-to state cannot be incorporated into the current cognitive map. This is the case in foraging paradigms [2, 3, 4] if decisions to forage (state-change decisions) take the observer to a previously unseen environment and therefore off the current cognitive map. For example, Kolling *et al.* [2] offered participants the choice on each trial of selecting within an offered set of stimuli (within-state choice), or ‘foraging’ — allowing the computer to exchange the whole set of stimuli for an unknown alternative set (state-change). In this paradigm the state-change decision involves evaluating the entire set of (currently) available options against an unknown set after foraging — in contrast to within state decisions which entail choosing within the current set of options. Therefore, it could be fairly clearly argued that when participants opt to change state, they are leaving the current cognitive map.

A softer distinction between within-state and state change decisions may be made in paradigms in which the participant alternates phases of engaging with one set of stimuli or behaviours, and disengaging or switching to a different set of stimuli or behaviours. For example, Stoll *et al.* [5] report a task in which at any given moment monkeys can either remain engaged in ‘work’ on a match-to-sample task, or disengage and ‘check’ the length of time remaining to a bonus reward. Since the monkeys knew what to expect when they switched to checking behaviour, it could be argued that the ‘work’ and ‘check’ tasks lie on a single cognitive map, but on the other hand since the ‘work’ and ‘check’ tasks involved different stimuli and associations, and the monkey could never be in the ‘work’ and ‘check’ tasks at the same time, the transition between them could be viewed as a state-change.

### Neural substrates of within-state and state-change decisions

Within-state decisions require a cognitive map of the environment (probabilistic stimulus-action-outcome associations) which is used to select the best course of action given the current motivational state of the agent. For example, a rat, which has learned that one lever leads to cheese and another lever leads to juice, can select the appropriate lever to obtain his currently preferred reward (cheese if hungry, juice if thirsty). The construction and use of such models depends on the orbital and lateral frontal cortex [6,7], ventromedial PFC [8] and hippocampus [9].

In contrast, state-change decisions require evaluating the currently available options as whole, against (possibly unknown) alternatives if a state-change is made. Global state-change decisions are particularly linked to the anterior cingulate cortex and adjacent dorsomedial prefrontal cortex, including the preSMA. The term dorsomedial prefrontal cortex is used in humans to encompass several areas which tend to co-activate in imaging studies. The rostral cingulate zone, which is connected to both subcortical reward centres including ventral striatum, and to motor outputs, includes the dorsal bank of the cingulate sulcus. Tissue dorsal and anterior to the ACC-proper, in the pre-SMA or anterior medial prefrontal cortex, tends to co-activate with ACC [2,10] and in some cases contains the strongest activity, even though activity extends into ACC proper (see [17] for review). Additionally, the human ACC may be more analogous to the dorsomedial prefrontal cortex in rats than to the rat ACC proper. Throughout the article we have used the term dorsomedial PFC to refer to activity peaking in, or extending strongly into, the dmPFC superior and anterior to the ACC. Where activity can be more specifically localized to the preSMA or ACC, we have used those terms.

dmPFC is active when agents decide to explore their environment. It is active in foraging decisions when agents seek a new state in the form of a new set of options [2]. It is also active when observers infer a change in state of the environment based on sensory data [10].

Multiple reasons for the link between dmPFC the decisions we refer to as state-change decisions have been proposed; here we will explore one particular aspect of these decisions, which is that they are made against the substrate of interval time. Interval time is defined as a continuous metric where the number of seconds elapsed between events is important, in contrast to ordinal time which concerns only the ordering of events but not the intervals between them, the distinction being analogous to the general distinction between interval and ordinal variables (see Coull *et al*. [11] for a review of the timing literature).

### Decision making in the temporal arena

State-change decisions are intrinsically temporal, in that the decision to be made is *when* (if ever) to engage or disengage with a state (i.e. an action, strategy or environment). This is in contrast to deciding *what* to do conditional upon a given state. We will argue that this temporal aspect of decision making poses unique computational challenges which the ACC is well placed to perform.

To decide when (if ever) to effect a state-change, decision makers need to weigh up the expected value of staying in the current global state or environment for a given length of time, against the expected value of state-change.

Formally, the expected value of remaining in a state, *s* (either the current one or an alternative) for time *T* is the integral over time from now to *T*, of the expected instantaneous reward rate $r_{s}\left(t\right)$ associated with being in state *s* at each future time t, over time.

(1) $$v_{s}\left(T\right)=\;\int_{0}^{T}r_{s}\left(t\right)dt$$

**Figure 1:**
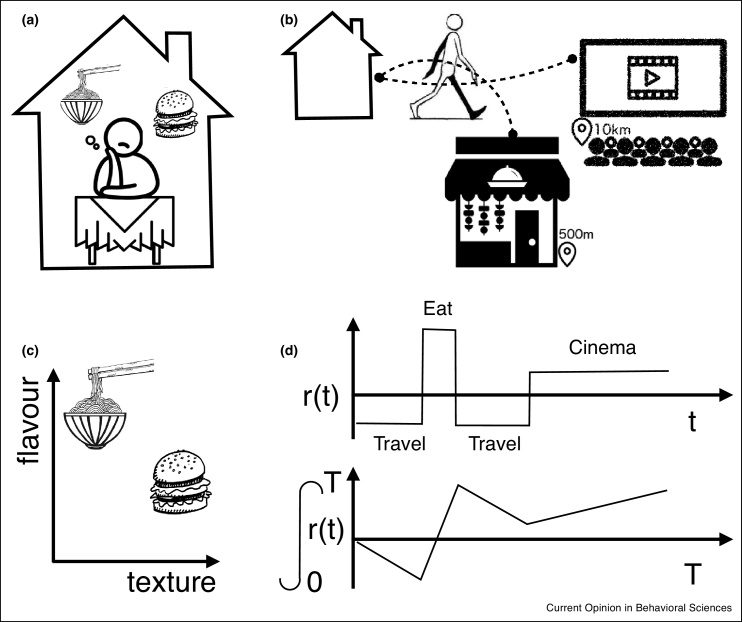
Within-state versus state-change decisions. **(a)** In within-state decisions, the agent’s task is to pick the best of the available options conditional on the current global state. For example given that I can order a burger or noodles for delivery, which do I want? **(b)** In state-change decisions the agent weighs the value of the whole global state (staying at home and ordering takeout) against the possible values of changed states (such as walking into town). **(c)** The computational challenge in within-state decisions case is to evaluate the options, which may involve evaluating each option over multiple attributes (flavour, texture) and weighting the attributes based on motivation. To achieve this the agent may use a model (here decision strategy) based on previous experience of the global state that represents the available options and their attributes as well as internal or hidden features such as hunger. **(d)** In state-change decisions, the agent determines what environment/global state to visit and how long for. Assuming the agent knows the expected instantaneous reward rate (reward) in an environment over time (top panel), it can compute the key decision variable: the expected integrated reward rate associated with a stay of a certain duration in that global state (bottom panel). This integrated reward rate is the sum of the instantaneous reward rate over time divided by time spent. Therefore, it is important to consider interval timing because the best available option is intrinsically tied to time itself.

The expected reward rate is expressed as a function of time as it is generally not a constant — for example if a state-change decision involves travelling to a new patch, the initial period after the state change will be associated with negative value as energy is expended travelling to the new patch. If the agent stays in the new patch for long enough, the subsequent positive reward rate will bring the expected value of the state-change back above zero. This concept is illustrated in Figure 1b.

### Temporally discrete vs continuous generative structures

The concept of expected reward rate is useful because it generalizes to ecological environments that are not neatly sliced into discrete trials.

In decision-making paradigms in the lab, tasks are usually divided into discrete trials with arbitrary inter-trial intervals. Therefore whilst such tasks may involve change over time, the maximal level of temporal structure is usually ordinal (i.e. the ordering of discrete trials rather than the number of seconds that have elapsed). The value of each candidate action could be modelled as the parameter of a (discrete) binomial distribution — the probability of reward if that option is picked.

In contrast, in a natural environment, there are no trials. For example, to evaluate the quality of a fishing spot, the key variable is the rate of fish capture over time. The value of the environment can be modelled as the parameter of a Poisson distribution, that gives the probability of capturing different numbers of fish, given the number of minutes spent.

The formalization of the generative structure of the environment (the mathematical rules by which rewards are generated) makes explicit the difference between trial-based (lab) environments and continuous-time (naturalistic) environments.

### Negative reward rate as a way of expressing costs

We have already seen that the value of being in a global state for a time period *t* can be formalized as the integral of reward rate over time. We will argue that this is also true for costs.

Action costs can be divided into opportunity and energetic cost. Opportunity costs are simply a negative contributor to the calculated benefit (i.e. they translate into a reduction in the expected amount of reward to be accrued). In ecological settings many opportunity costs are intrinsically temporal, for example travel time in patch leaving scenarios [3,12], and handling time in foraging [4,12], both incur costs as the agent is not harvesting reward during these time periods. These translate into periods of time during which the reward rate *r(t)* is zero. Thus they bring down the overall, integrated value of being in a global state.

Energetic costs (such as energy expended in pursuing prey) cannot be directly calculated in terms of forgone reward or time wasted. However, energetic responses can be very naturally expressed as a function of interval time, i.e. a rate. The frequency of responses and the effort expended can be combined into a single metric of response vigour [13,14], which determines the rate of energy expenditure.

Once the costs and benefits of a state are expressed in terms of rate, the complex decision space of benefits, delays, effort and reward reduces to a simple difference of mean reward captured minus mean costs incurred, both per unit time [15]. The problem of maximizing energetic gain then reduces to maximizing this difference.

Importantly, this single metric of environmental value, as reward rates minus rate of energy expenditure, allows an agent to make global decisions between qualitatively different kinds of environments and subsequent actions, such as comparing different potential evening activities (Figure 1). The use of ecological paradigms in which agents have the option to make such global decisions, in the context of real time and real costs, may therefore be important for understanding the role of dmPFC.

### Medial prefrontal cortex is well placed to make decisions using interval time

Medial prefrontal cortex is particularly involved in a range of decisions that we would group as state-change decisions, including decisions to explore [4], forage [2], switch action strategy [16] or update one’s internal model of the environment [10] some of these tasks have been shown to engage ACC proper (tissue in the dorsal bank of the cingulate sulcus) whilst others have more strongly engaged regions dorsal and anterior to ACC, with similar but not identical connnections and functionality — for review see [17]. Many reasons for its involvement have been proposed but here we address specifically the role of medial prefrontal cortex in relation to interval timing, which we have argued above is particularly important for engage-disengage decisions.

**Figure 2:**
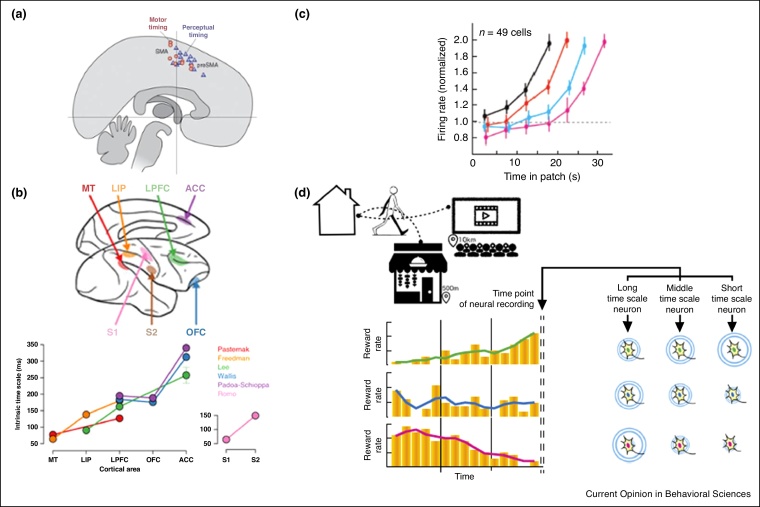
Medial prefrontal cortex and time. **(a)** Meta analysis showing peak activity in a series of fMRI studies related to interval timing judgments. Many of these tasks activate dmPFC, with peaks in SMA and pre-SMA. From [11]. **(b)** Neurons in ACC additionally appear to have a large neural integration time constant (intrinsic timescale), compared to most other brain regions, allowing change in reward rate over time to be estimated. Here intrinsic timescales were estimated from different recording studies and estimates of degree of auto-correlation over time. From [28]. **(c)** Differing timescales could translate into different learning speeds. In fact, if learning rates are fit on individual neurons (example of one such fits shown as black line) a diversity of learning rates emerge in ACC. From [31]. **(d)** However, ACC does not passively track time or rewards, during patch leaving decisions ACC neuron firing ramps up consistently until animals leave independently of number of seconds past (each coloured line indicates a different travelling time, which delays leaving decisions), proving that reward related signals must be discounted by the value of the global state, which decreases with increased travelling times. From [4]. **(e)** Combining the existence of different reward timescale neurons and global state sensitive decision signals, ACC could allow an agent to predict the future trajectory of global states/environments, important for global decision making in general and timing decisions specifically. From [17].

Experimental paradigms that directly probe interval timing, such as interval production and discrimination, are associated with activity in medial prefrontal cortex including ACC and adjacent preSMA (Figure 2a and [18,19]). Indeed, if interval timing is viewed a behaviour in itself, the frontal-striatal circuit (medial PFC and connected putamen) seems to be its location in the brain; interval judgements selectively engage this circuit even when other task aspects such as working memory and the need to integrate evidence over time are controlled [19].

Medial frontal cortex and connected regions of striatum display temporally specific responses including notably ramping activity leading up to the end of a timed interval (ERPs in humans [20]). Single cells in macaques [21] and rats [22] show neural selectivity for the initiation of different wait-intervals [21]. An influential computational model of timing, the beat frequency model, postulates a set of oscillators with different frequencies, combinations of which would ‘beat’ at different behaviourally relevant intervals [23]. The striatal-medial frontal circuit is thought to be a likely substrate for such an oscillator–accumulator network, with striatum playing the role of the oscillator and medial frontal cortex, the accumulator [24].

Dopaminergic inputs, and dopaminergic modulation of cortico-striatal interactions, may play a key role in the timing functions of medial PFC. Interval timing judgements are strongly influenced by dopaminergic tone. Parkinson’s disease patients judge intervals to be longer when off dopamine agonist medication than on [25], possibly due to the distorted storage in memory of timed intervals. In healthy subjects, dopamine depletion affects interval timing judgements, and the extent of the effect is proportional to the change in fMRI signal in putamen and preSMA during interval encoding [26].

However, dopamine is also important in terms of reward processing, and effortful action. It has already been proposed that dopamine provides a natural link between average reward rate and response vigour in that a higher reward rate results in more dopamine, but conversely dopamine also invigorates behavioural output [13]. Thus reward rates, and simply the rate of time passing are intrinsically linked by dopamine.

ACC is a major cortical target of dopaminergic projections and is thus well placed to integrate this rate-related information with knowledge about states and possible actions.

### Decision-making and projecting over time

To maximize the difference between reward rate and cost rate, it is not sufficient to evaluate the current rate of reward and cost. Instead, the choice to engage or disengage depends on the integral of the future expected reward rate (minus cost rate), over the time intended to be spent in a chosen global state (Eq. (1), Figure 1b + d). In natural environments reward rates are rarely static — for example patches of fruit may be depleted by consumption or by seasonal change, whilst the location of animal prey changes over time due to diurnal and seasonal migration.

ACC is equipped to project the future rate of reward. ACC is sensitive not only to current reward, but to trends in reward, such that if current reward is lower than the past average, activity increases which in turn predicts disengagement from the state [27]. Neurons in ACC furthermore have one of the highest integration time constants in neocortex (Figure 2b; [28]), meaning that they can track slow changes in reward rate.

ACC is sensitive to the rate of change of the environment [29] and is able to track change over multiple timescales in parallel, as it contains a functional gradient in which anterior tissue is more sensitive to rapid change [30]. In monkeys, neurons with many different time constants for reward memory (Figure 2c) and a wide range of input integration time constants ranging from miliseconds to tens of seconds, have been observed in ACC (Figure 2b; [31]). The relative activity in short timescale cells compared to long timescale ones could be used to calculate trends in reward rate (Figure 2e).

In addition to temporal trends in reward rate, natural environments contain structured variations in reward rate over time. The most obvious is exponential decay: Consumption of a resource at a rate proportional to the remaining quantity of the resource results in the characteristic exponential decay curve. Single neurons in ACC have been shown to follow such patterns to ramp up to a patch leaving decision (Figure 2d; [4]). However, some resources follow oscillatory (diurnal or seasonal) patterns and complex, arbitrary patterns are also possible. Whether such structured information is used by ACC to predict future reward rate is unknown.

### The ACC does not only track reward rate

We have argued that the ability to work in the context of interval time is an important aspect of the ACC’s role in decision-making. Specifically, ACC calculates the projected average reward rate (minus cost rate), which is a unitary decision variable driving agents to remain in a state or disengage. This is compatible with findings that ACC is active during exploratory choices [32] and that ACC neurons are active when feedback indicates that a change of behaviour would be advantageous [33].

**Figure 3:**
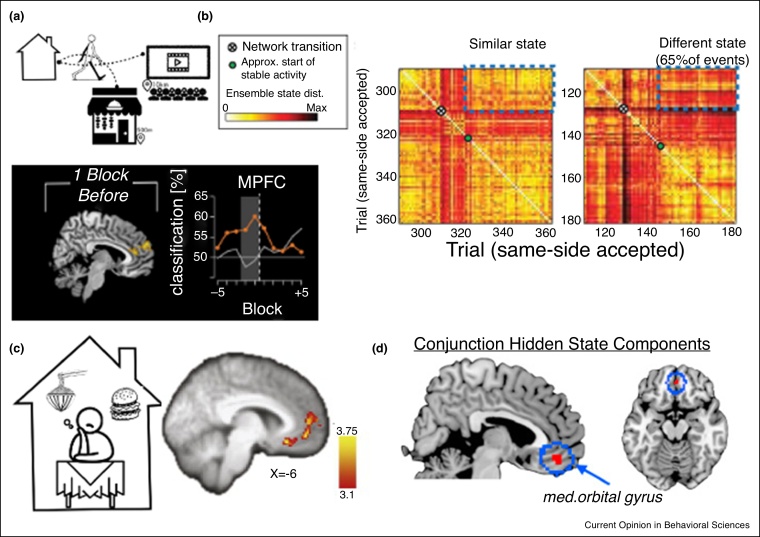
**(a)** Global state change decisions can lead to important resets of neural dynamics to enable new learning and further adaptation of behavioural strategies and future choices. In humans, dmPFC appears to predict future behavioural insights and internal strategy shifts at least one block prior to those changes being apparent in the choices. In an ROI analysis gradual increase of such representations across several blocks are apparent (lower panel). From [39]. **(b)** In rats, abrupt changes in behavioural strategies lead to overall neural network resets generating a new neural state altogether. This is particularly apparent using representational similiarity analysis on a population of ACC neurons across trials that include stable states interrupted by periods of behavioural change. The cross indicates the onset of network transition and the green dot the beginning of a new and often distinct stable state. From [16]. **(c)** On the other hand, OFC is particularly important for local decisions, exploiting a specific general model/decision strategy. Particularly ventromedial prefrontal cortex (vmPFC), a sub-part of larger OFC, is important for integration of value across dimensions when making decisions, scaling its activity with the relative value of the chosen option (Boorman et al 2009) (see Hunt *et al.*, for a bio-physical model of the mechanism and precise temporal predictions). From [40]. **(d)** Additionally, vmPFC also carries information about hidden states, at least when they are important for making context sensitive accurate classification decisions. From [36].

However, the information in ACC is rich, and certainly not limited to a single decision variable. ACC represents not only the pressure to disengage from the current state, but information about the current state itself. ACC is connected to the motor system and has access to the current action plan: ACC cells are sensitive to the action chosen [34], and the adjacent preSMA, very often co-activated with ACC in imaging studies, is central to the process of self-initiated action [35]. Patterns of activity across ACC may represent not only the current action, but a stable behavioural state or strategy. In one particularly clear study [16], the pattern of activity across multiple units in rat medial prefrontal cortex remained stable as long as behavioural strategy was stable, but became chaotic when the rat was unsure about the correct behavioural strategy (exploring) and settled to a new pattern once a new stable behavioural state was reached (Figure 3b). Furthermore, ACC may represent candidate future states as well as the current state: In a perceptual judgement task in which participants voluntarily switched strategies part way through, dmPFC was shown to encode the switched-to strategy in the run-up to switching [36] (Figure 3a).

Interestingly, though, strategy representations in ACC appear to be intrinsically temporal in that they are *temporary*. ACC has stable patterns of activity during stable strategies, but if the animal leaves a strategy and then later returns, the pattern of activity is different (Figure 3b; [16,37]). Furthermore, neurochemical manipulations of ACC that cause animals to switch from strategic to random behaviour, apparently by inactivating the strategy representation in ACC, do not prevent a behavioural return to strategy later on [38]. This suggests that some representation of the abandoned strategy is retained elsewhere in the brain and therefore ACC cannot be the unique seat of generalized strategy representations.

Taken together with the evidence that ACC tracks the need for behavioural change, one possible interpretation is that the purpose of strategy representations in ACC is evaluative — rather than storing stable knowledge about possible behavioural strategies, the ACC contains mutable representations that implicitly represent the quality of the current strategy and are thus temporally specific. In other words, it might signal a unique combination of reward history, current strategy and state that is dynamically dependent on when it is encountered. When the same strategy is implemented a second time the agent will have transitioned through several different behaviours that changed the current behavioural and neural state as well as history, thus making the current strategy assessment distinct.

### Different imperatives for decision-making

We have argued that in ecological contexts agents need a sense of how good the current environment or strategy is *right now* to make the temporal decision of when to disengage, and that this is the role of ACC. This however, is the direct opposite of another imperative for efficient decision making of being able to create general and time-insensitive representations in order to allow generalization and through that more sophisticated model-based learning.

If the purpose of a representation is to capture how good the environment is *right now,* when an agent leaves an environment and later revisits it, the state of its model at the last point that environment was seen is irrelevant — so the representation may change from one episode to the next. This could explain why ACC representations appear to be inconsistent across behavioural episodes.

In contrast, if the purpose of a representation is to make the best choice conditional on the state, the model should integrate experience over multiple past encounters with that state. OFC contains a state space representation of this type: it represents one’s location in a state space in a way that is stable over time. If an agent returns to the same position in state space, the pattern of activity in the agent’s OFC is the same [36,41]. These detailed models of action-outcome associations conditional on state mean that it can implement specific models of the world (e.g. decision strategies) allowing it to integrate across different value dimensions to extract subjective choice value (Figure 3c; [40,42]) and even use hidden states to infer correct choices (Figure 3d; [36]).

In other words, the different types of calculation performed in ACC and OFC fit them to fulfil different but complementary behavioural goals. While ACC might be well placed to make global decisions taking into account current rates, context and alternatives, OFC might allow for optimal local decisions by generating a generalized sense of subjective and local preference.

### Conclusion

We have drawn a distinction between state-based decisions, in which an agent chooses the best course of action given its circumstances, and state-change decisions, in which an agent decides whether and when to change its circumstances. We have argued that the two types of decisions require neural representations of the current state with different qualities and may be performed in different brain areas.

State-based decisions require a stable map of the state space, with precise and accurate knowledge of the contingencies between stimuli, actions and outcomes and the structure of the environment. Such models may be constructed over multiple, temporally separated learning episodes and should remain stable unless the actual contingencies within a state change permanently. Thus state-based decisions require models, which are precise in contingency but general in time. They would allow precise local choices among a defined set and given a specific decision strategy, but not necessarily the more global decision of whether engaging with such a choice or whether the overall context is worth it, given ones own internal and external environment.

State-change decisions on the other hand require knowledge of the current and projected reward rate associated with the state as a whole and the value of overall decision strategies compared to all possible alternative environments. The substrate of these decisions is intrinsically temporal (deciding when to act) and the decision variable (reward rate minus cost rate) is also intrinsically temporal. In as far as the state itself is represented, there is some evidence that the state representation is stable only on the timescale of state changing itself and does not represent a lasting representation of contingencies in a given state. Thus such models are precise in time and global in nature but are probably not the primary representation of local within-state contingencies.

## Conflict of interest statement

Nothing declared.

## References and recommended reading

Papers of particular interest, published within the period of review, have been highlighted as

• of special interest
•• of outstanding interest
